# Olaparib and temozolomide in desmoplastic small round cell tumors: a promising combination in vitro and in vivo

**DOI:** 10.1007/s00432-020-03211-z

**Published:** 2020-04-11

**Authors:** Anke E. M. van Erp, Laurens van Houdt, Melissa H. S. Hillebrandt-Roeffen, Niek F. H. N. van Bree, Uta E. Flucke, Thomas Mentzel, Janet Shipley, Ingrid M. E. Desar, Emmy D. G. Fleuren, Yvonne M. H. Versleijen-Jonkers, Winette T. A. van der Graaf

**Affiliations:** 1 Department of Medical Oncology, Radboud University Medical Center, P.O. Box 9101, 6500 HB Nijmegen, The Netherlands; 2 Department of Pathology, Radboud University Medical Center, P.O. Box 9101, 6500 HB Nijmegen, The Netherlands; 3 Dermatopathology Bodensee, Friedrichshafen, Germany; 4 Sarcoma Molecular Pathology Team, Divisions of Molecular Pathology and Cancer Therapeutics, Institute of Cancer Research, London, UK; 5 Children’s Cancer Institute Australia, Lowy Cancer Research Centre, University of New South Wales, Sydney, NSW Australia; 6 Department of Medical Oncology, The Netherlands Cancer Institute-Van Leeuwenhoek, 1066 CX Amsterdam, The Netherlands

**Keywords:** Desmoplastic small round cell tumor (DSRCT), Poly(ADP-ribose) polymerase (PARP), Schlafen-11 (SLFN11), Olaparib, Temozolomide, Combination treatment

## Abstract

**Purpose:**

Desmoplastic small round cell tumors (DSRCTs) are highly malignant and very rare soft tissue sarcomas with a high unmet need for new therapeutic options. Therefore, we examined poly(ADP-ribose) polymerase 1 (PARP1) and Schlafen-11 (SLFN11) expression in DSRCT tumor tissue and the combination of PARP inhibitor olaparib with the alkylating agent temozolomide (TMZ) in a preclinical DSRCT model.

**Methods:**

PARP1 and SLFN11 have been described as predictive biomarkers for response to PARP inhibition. Expression of PARP1 and SLFN11 was assessed in 16 and 12 DSRCT tumor tissue samples, respectively. Effects of single-agent olaparib, and olaparib and TMZ combination treatment were examined using the preclinical JN-DSRCT-1 model. In vitro, single-agent and combination treatment effects on cell viability, the cell cycle, DNA damage and apoptosis were examined. Olaparib and TMZ combination treatment was also assessed in vivo.

**Results:**

PARP1 and SLFN11 expression was observed in 100% and 92% of DSRCT tumor tissues, respectively. Olaparib treatment reduced cell viability and cell migration in a dose-dependent manner in vitro. Drug synergy between olaparib and TMZ was observed in vitro and in vivo. Combination treatment led to a cell-cycle arrest and induction of DNA damage and apoptosis, even when combined at low dosages.

**Conclusion:**

We show high PARP1 and SLFN11 expression in DSRCT tumor material and antitumor effects following olaparib and TMZ combination treatment in a preclinical DSRCT model. This suggests that olaparib and TMZ combination treatment could be a potential treatment option for DSRCTs.

**Electronic supplementary material:**

The online version of this article (10.1007/s00432-020-03211-z) contains supplementary material, which is available to authorized users.

## Introduction

Desmoplastic small round cell tumors (DSRCTs) are very rare soft tissue sarcomas (STS) with an incidence rate of 0.2–0.5/million. DSRCTs are most often seen in the abdominal cavity of predominantly adolescent and young adult males (Lettieri et al. 2014). Patients often present with extensively disseminated disease at diagnosis, and their tumors are characterized by a t(11;22)(p13;q12) genetic translocation resulting in the oncogenic fusion protein EWS–WT1. The treatment of DSRCTs consists of intensive combination chemotherapy, when possible, surgery—sometimes combined with hyperthermic intraperitoneal chemotherapy (HIPEC)—and on indication radiotherapy, including whole abdominal irradiation. These treatments can be toxic and despite the fact that a small subset of patients shows a good response to treatment, this response is relatively short lasting (Hayes-Jordan et al. 2016). Second-line treatment for patients with recurrent disease that have been used are vascular endothelial growth factor (receptor) (VEGF(R))-, mammalian target of rapamycin (mTOR)-, and platelet-derived growth factor receptor (PDGFR)-based targeted therapy. These treatments can again induce favorable, however short-lived, responses (Chen and Feng 2019; Italiano et al. 2013; Menegaz et al. 2018; Tarek et al. 2018; Thijs et al. 2010). Overall, treatment results in a 5-year overall survival (OS) rate of 15–25%, which shows the high unmet need for novel treatments in DSRCTs (Bent et al. 2016; Subbiah et al. 2018).

Given the need for novel treatments, we looked towards targeted therapy directed against the DNA damage response (DDR) machinery. The DDR network appears to be a potential target for DSRCTs since the EWSR1–WT1 translocation already involves two DDR network proteins (Gorthi and Bishop 2018; Oji et al. 2015) and the recent detection of multiple mutated genes belonging to the DDR network (Devecchi et al. 2018) by whole-exome sequencing of 6 DSRCT samples. We hypothesize that the presence of aberrations in the DDR pathway will most probably make DSRCTs more vulnerable for additional inhibition of the DDR system.

PARP1 is a key enzyme in the base excision repair (BER) of single-strand DNA breaks (SSBs). PARP1 senses SSBs, and recognition leads to the binding of PARP1 to the DNA and synthesis of poly (ADP-ribose) (pADPr) chains. Both PARP1 and pADPr chains are involved in the recruitment of DNA repair proteins, and pADPr chains mediate the release of PARP1 from the DNA to ensure access of the repair proteins to the damaged site. Inhibition of PARP1 leads to an accumulation of SSBs and trapping of PARP1 to the DNA. Inadequate repair of the SSBs causes double-stranded breaks (DSBs) during DNA replication, and PARP1 trapping prevents the formation of replication forks. Both these effects are lethal to the cell (Lord and Ashworth 2012, 2017). In ES, PARP1 expression is suggested to be regulated by the fusion proteins EWS–FLI1 and EWS–ERG, and a feedback loop is present in which PARP1 promotes the transcriptional activity of the fusion proteins. Single-agent efficacy of the anti-PARP inhibitor olaparib was indeed shown to be dependent on the presence of a EWS fusion since fusion-negative cell lines were not sensitive to treatment (Brenner et al. 2012). As such, PARP inhibition could also have therapeutic potential in the EWS fusion-positive DSRCTs. In addition to PARP1 expression, Schlafen-11 (SLFN11) expression is also regulated by the EWS fusion and has recently been suggested as a biomarker for response to PARP inhibitor-based treatment (Lok et al. 2017; Pietanza et al. 2018; Tang et al. 2015). SLFN11 induces a lethal replication block in cells under replication stress (Murai et al. 2018). Therefore, SLFN11-positive cells are more efficiently killed by treatments that cause replication stress like PARP inhibition (Murai et al. 2019).

Here, we examined PARP1 and SLFN11 expression in clinically derived DSRCT tissue (*n* = 16) and PARP inhibitor-based treatment effects in a DSRCT model. Since previous research showed that single-agent PARP-targeted treatment did not elicit high responses in ES patients (Choy et al. 2014; Vormoor and Curtin 2014) and combination treatment using the alkylating agent temozolomide (TMZ) led to a synergistic effect in ES in vitro, a complete tumor regression and reduction of lung metastases in ES in vivo*,* and a clinical trial is currently examining the combination (NCT01858168), we examined the combined effect of PARP inhibitor olaparib and TMZ in DSRCTs (Brenner et al. 2012; Engert et al. 2015; Gill et al. 2015; Ordonez et al. 2015; Smith et al. 2015; Stewart et al. 2014). TMZ has been described in a few case reports to be administered to DSRCT patients in combination with irinotecan. Umeda et al. administered TMZ at 120 mg/m^2^ during the first 5 days of four 28-day cycles. A partial response of the bone metastasis and pineal body was observed; whereas, the cerebellar lesions showed stable disease (Umeda et al. 2016). Hayes-Jordan et al. presented 2 cases that were treated with TMZ and irinotecan (6 cycles), one showed a decrease of tumor mass and the other showed stable disease (Hayes-Jordan et al. 2007).

In another case report, temozolomide was administered in combination with irinotecan (12 cycles) to a child with DSRCT after extensive neoadjuvant chemotherapy treatment, cytoreductive surgery and hyperthermic peritoneal perfusion with cisplatin. Afterwards, abdominal radiation with simultaneous temozolomide (100 mg/m^2^/day × 5) was given. Due to the extensive multimodal treatment, the specific effect of temozolomide could not be filtered out (Aguilera et al. 2008).

The combination of TMZ with olaparib has not been described for DSRCTs. Current clinical examination of combination treatment often combines a maximal tolerated dose (MTD) of each compound; however, drug synergy between compounds might make it possible to reduce the dosage necessary to generate antitumor effect. Since the use of low dosages may be able to reduce the level of toxicities encountered in patients, we specifically examined low-dose combination treatment regimens.

## Materials and methods

### PARP1 and SLFN11 expression in patient-derived DSRCT tumor tissue

Clinically derived DSRCT tumors were assessed for PARP1 (16/16) and SLFN11 (12/16) expression by immunohistochemistry (IHC). Table 1 shows the patient characteristics. PARP1 and SLFN11 IHC were performed on 4-µm-thick, formalin-fixed, paraffin-embedded (FFPE) whole-slide tissue sections and a tissue microarray (TMA) (core size 1 mm) of DSRCT tumor material. Tonsil tissue and lymphocytes served as a positive control for PARP1 and SLFN11, respectively (Fig. S1). Sections were deparaffinized in xylol and rehydrated through a graded ethanol into water series. Antigen retrieval was performed by heating the slides in EDTA buffer, pH 9 for 10–20 min at 100 °C. Endogenous peroxidase activity was blocked with 3% H_2_O_2_ in distilled water for 10 min at room temperature (RT). Subsequently, sections were incubated with monoclonal rabbit anti-PARP1 antibody (1/800, clone E102, Abcam) or monoclonal rabbit anti-SLFN11 antibody (1/100, clone D8W1B, Cell Signaling Technology) in antibody diluent in a humidified chamber overnight at 4 °C. Next, tissue sections were incubated with poly-HRP-GAMs/Rb IgG (ImmunoLogic) in EnVision™ FLEX Wash Buffer (Dako) (1:1) for 30 min at RT. Antibody binding was visualized using the EnVision™ FLEX Substrate Working Solution (Dako) for 10 min at RT. Finally, slides were counterstained with haematoxylin, dehydrated and coverslipped. Slides were scored for PARP1 expression by two independent observers and consensus nuclear scores were given as negative (−) or positive (+) with a minimum cut-off at 50% of tumor cells, based on the paper of Grignani et al. (2018). Similar scoring methods were used for SLFN11. The study was performed in accordance with the Code of Conduct of the Federation of Medical Scientific Societies in the Netherlands.

**Table 1:** Patient characteristics and PARP1/SLFN11 expression in DSRCT tumor tissue

Tumor type	Characteristics	*N* (%)
DSRCT (*n* = 16)	Gender	
	Male	11 (69)
	Female	5 (31)
	Age at diagnosis	
	< 18 years	4 (25)
	≥ 18 years	12 (75)
	Translocation	
	EWSR1–WT1	16 (100)
	Metastases	
	Yes	10 (63)
	Unknown	6 (38)
	Primary/post-treatment resection	
	Primary	10 (63)
	Post-treatment	6 (38)
	Follow-up data available	
	OS	13 (81)
	EFS	2 (13)
	PARP1 expression	
	≥ 50% cells	16 (100)
	< 50% cells	0 (0)
	SLFN11 expression (*n* = 12)	
	≥ 50% cells	11 (92)
	< 50% cells	1 (8)

*DSRCT* desmoplastic small round cell tumor, *n* number of tumor tissues, *y* year of age, *OS* overall survival, *EFS* event-free survival, *PARP1* poly(ADP-ribose) polymerase 1, *SLFN11* Schlafen-11

### Cell lines, cell culture and compounds

The only established DSRCT cell line, JN-DSRCT-1 (*EWSR1-WT1*), was generously provided by Dr. Janet Shipley (Institute of Cancer Research, UK). JN-DSRCT-1 was cultured in DMEM/F12 GlutaMAX™ medium (Gibco, ThermoFisher, Breda, NL) supplemented with 10% fetal calf serum (Gibco) and 1% penicillin–streptomycin (Lonza, Breda, NL). Cells were cultured in a humidified atmosphere of 5% CO_2_/95% air at 37 °C. PARP inhibitor olaparib and TMZ were purchased from SelleckChem (Munich, Germany) and were diluted in DMSO for in vitro experiments. TMZ and olaparib were diluted in 10% DMSO in saline (intraperitoneal injection) and in 0.5% hydroxypropyl methylcellulose/0.2% Tween-80 in sterile water (oral gavage) for in vivo use, respectively.

### Cell viability and wound healing assay

Cell viability was assessed by MTS assays. All cells were seeded at 5000 cells per 100 μl/well. Cells were allowed to adhere and treated with varying drug concentrations for 120 h, based on the estimated growth rate of JN-DSRCT-1 cells. MTS solution (CellTiter 96 Aqueous Solution Cell Proliferation Assay, Promega, WI, USA) was added (10 µl) and plates were incubated for 2 h at 37 °C. Extinction was measured at 490 nm (iMark Microplate Absorbance Reader, Bio-Rad, CA, USA). IC_50_ values were calculated using GraphPad Prism Version 5.03 software.

Effects of treatment on cell migration were assessed by wound healing assays as previously described (van Erp et al. 2017). Cell migration is depicted in relative gap size: gap size at *t*_N_/gap size at *t*_0_ (*t*_N_ = hours of treatment, *t*_0_ = start of treatment). Differences in gap size were analyzed by 2-way ANOVA with Bonferroni posttest, *p* value < 0.05 was considered significant (*< 0.05, **< 0.01, ***< 0.001).

### Drug synergy and combination index

Drug synergy of combined olaparib and TMZ treatment was assessed as previously described (van Erp et al. 2017). All drug concentrations were simultaneously combined in a non-constant ratio, and the combination index (CI) and dose reduction index (DRI) were calculated using CompuSyn software. In general, combinations with a CI value < 1.0 are considered synergistic; however, a distinction in the level of drug synergy can be made (Table 4; Chou 2006). Two distinctions were relevant for this paper: CI between 0.7–0.9: slight to moderate synergism, and CI between 0.3–0.7: synergism. Differences in cell viability following combination treatment were analyzed by 2-way ANOVA with Bonferroni posttest using GraphPad Prism Version 5.03 software, *p* value < 0.05 was considered significant (*< 0.05, **< 0.01, ***< 0.001).

### Cell cycle, Western Blot and apoptosis analysis

Cell-cycle analysis was performed using propidium iodide (PI) flow cytometry. Cells were treated for 24 h with vehicle, 1.25-µM olaparib, 25-µM TMZ (low-dose TMZ), 100-µM TMZ (high-dose TMZ) or simultaneous 1.25-µM olaparib and 25-µM (low-dose) or 100-µM (high-dose) TMZ combination treatment. Cells were collected and incubated overnight on ice at 4 °C in a PI solution [sodium-citrate dihydrate solution (1 g/l), RNAse A (0.1 mg/ml), PI (20 µg/ml), Triton-X (0.1%)] and the cell cycle phases were assessed using the CytoFlex flow cytometer (Beckman-Coulter, CA, USA) and FlowJo version 10.0. Experiments were repeated in triplicate and *p* values were calculated by 2-way ANOVA with Bonferroni posttest.

Western Blot analysis was performed as previously described (van Erp et al. 2017). Monoclonal rabbit anti-PARP1 (PARP1, 1:2000; cat. #9542), anti-caspase-3 (casp3, 1:1000; cat. #9662), anti-phosphorylated H2AX (ser319) (γH2AX, 1:1000, cat. #9718), anti-phosphorylated Chk1 (pChk1 Ser317/345, 1:1000; cat. #12302/2348) and anti-phosphorylated Chk2 (pChk2 Thr68, 1:500; cat. #2197) were purchased from Cell Signaling Technology (Danvers, MA, USA). Loading control monoclonal mouse anti-α-tubulin (1:1000, cat. #A11126) or anti-GAPDH (1:10,000, cat. #Ab8245) were purchased from Thermo Scientific (Breda, NL) and Abcam (Cambridge, UK), respectively.

The level of apoptotic cells was measured using the Annexin-V/PI double-staining apoptosis assay (Biovision Cat. #1001–200, CA, USA). Cell culture medium was supplemented with CaCl_2_ (final concentration 15 mM) and the cells were subsequently incubated with Annexin-V-FITC and PI. The number of apoptotic cells was measured using the CytoFLEX flow cytometer and the percentage of early (Annexin-V positive/PI negative) and late (Annexin-V/PI positive) apoptotic cells was calculated using FlowJo version 10.0.

### Caspase inhibition and RT-qPCR

To examine caspase-dependent apoptosis, cell viability was assessed in the absence and presence of 50-µM pan-caspase inhibitor zVAD.FMK (MedChemExpress, Sollentuna, Sweden). Cell viability was assessed by MTS assay. Experiments were repeated in duplicate and *p* values were calculated by Student’s *t* test using GraphPad Prism Version 5.03 software, *p* value < 0.05 was considered significant (**< 0.01).

Gene expression was assessed by RT-qPCR. Cells were treated with 24-h single-agent or simultaneous combination treatment. Trizol-based RNA isolation was followed by cDNA synthesis (iScript cDNA synthesis kit, BioRad, CA, USA) and SYBR green-based qPCR. Primer sequences for the pro-apoptotic proteins BAX, BAK and BID are described in Table S1.

### In vivo therapy experiment

Male CB-17/lcr-Prkdcscid/Rj SCID mice (6–8 weeks) were subcutaneously injected with 5 × 10^6^ JN-DSRCT-1 cells in a 1:1 culture medium: Matrigel^®^ Matrix (Corning, NY, USA) solution. The mice were randomly divided into the four treatment groups once the tumor size reached 0.3 cm^3^. Treatment consisted of 28 days of either vehicle (*n* = 5), single-agent olaparib (50 mg/kg twice daily; *n* = 5), single-agent TMZ (25 mg/kg for 5 days twice daily with 2 days rest; *n* = 5) or olaparib and TMZ combination treatment (twice daily 50 mg/kg olaparib with 5 days twice daily 25 mg/kg TMZ; *n* = 4). All compounds were color coded ensuring blinding throughout the experiment. Tumor growth was monitored by caliper measurements in three dimensions [length (*l*), width (*w*) and height (*h*); all maximum diameter] twice weekly. Tumor size was calculated using the formula: 4/3*π* × l/2 × *w*/2 × *h*/2. Mice were euthanized on day 28. If the relative tumor volume was < 50% of the start volume at day 28, the experiment was extended for half of the affected mice for another 28 days. The mice were kept without further treatment to determine the duration of tumor regression upon treatment withdrawal. Tumor sizes are depicted as relative tumor volume (RTV) ± standard error of the mean (SEM) [RTV = tumor volume at any time (*V*_*t*_)/tumor volume at *t* = 0 (*V*_*t*0_)]. Differences in tumor volume were calculated by Student’s *t* test using GraphPad Prism Version 5.03 software, *p* value < 0.05 was considered significant (*< 0.05, **< 0.01, ***< 0.001). After excision of the tumor, H&E staining was used to assess tissue damage and tumor vasculature post-treatment.

## Results and discussion

### PARP1 and SLFN11 expression in patient-derived DSRCT tumor tissue and single-agent olaparib treatment effects in JN-DSRCT-1 cells

To examine the presence of PARP1 in DSRCT tumor tissue, PARP1 expression was assessed by immunohistochemistry. Sixteen DSRCT samples were examined, and nuclear PARP1 was present in > 50% of the tumor cells in 100% of the samples (Table 1/Fig. S1). Pignochino et al. previously showed in a variety of sarcomas that high PARP1 expression correlated with PAR activity and sensitivity to PARP inhibitor-based combination treatment in vitro (Pignochino et al. 2017). In addition, patients with tumors expressing high PARP1 experienced a significantly higher progression-free survival rate post-olaparib and trabectedin combination treatment compared to patients with low-level PARP1 expression in the tumor (Grignani et al. 2018). In addition to PARP1, the expression of SLFN11 has been suggested to predict a response to PARP-based treatment in ES (Lok et al. 2017; Pietanza et al. 2018). The expression of SLFN11 was examined in 12 DSRCT tumor tissues and showed expression in > 50% of the tumor cells in 11/12 tumor samples (Table 1/Fig. S1). The high PARP1 and SLFN11 levels in virtually all clinical DSRCT tumor specimens prompted us to examine the preclinical effect of PARP inhibitor olaparib with alkylating agent TMZ combination treatment in a preclinical DSRCT model.

**Fig. 1:**
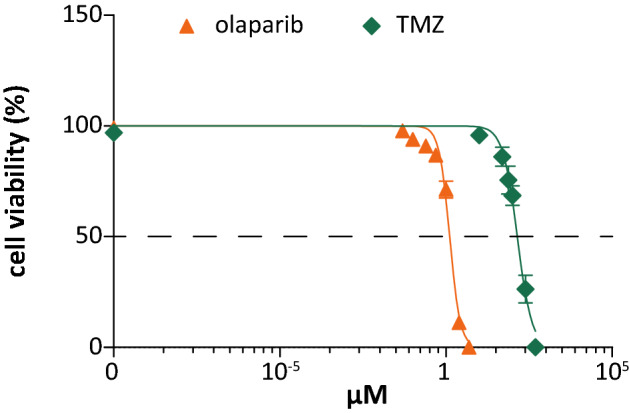
Single-agent olaparib treatment effects in DSRCT cells. Cell viability (%) following single-agent olaparib and TMZ treatment in the JN-DSRCT-1 cell line

In vitro, JN-DSRCT-1 cells showed an IC_50_ value equal to 1.38 ± 0.2 µM and 166 ± 93 µM following olaparib or TMZ treatment, respectively (Fig. 1a). The relatively high IC_50_ value of TMZ was comparable with that of the Ewing sarcoma cells we tested (ES7 143 µM and ES8 234 µM) and those tested by Engert et al. (> 200 µM) (Engert et al. 2015).

### Olaparib and TMZ combination treatment

**Fig. 2:**
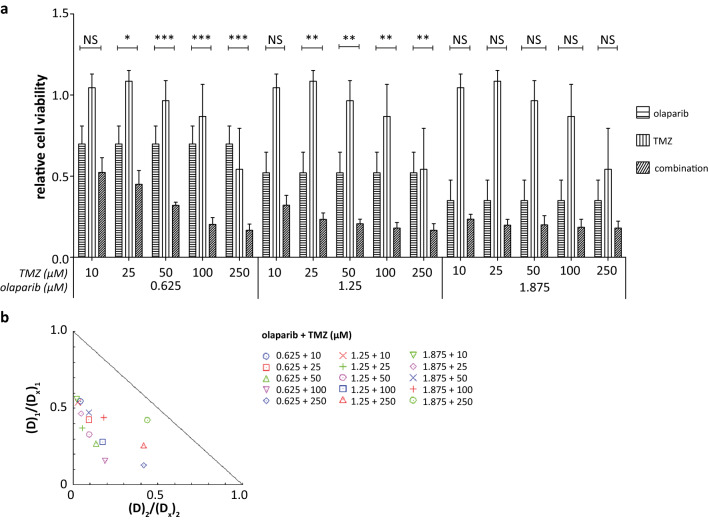
Olaparib and TMZ combination treatment in DSRCT cells. **a** Relative cell viability following non-constant ratio olaparib and TMZ combination treatment in the JN-DSRCT-1 cell line. Cell viability of the vehicle-treated cells is represented by the dotted line. Differences in cell viability assessed by 2-way ANOVA with Bonferroni posttest. *p value < 0.05, **p value < 0.01, ***p value < 0.001. Asterisks represent a significant difference between the combination treatment and both single-agent treatments. **b** Isobologram of the non-constant ratio combination treatment with increasing concentrations of olaparib (0.625; 1.25; 1.875 µM) and TMZ (10; 25; 50; 100; 250 µM). The *X*- and *Y*-axis of the isobologram represent the fraction of the portion of the drug in the combination treatment (*D*_1_ + *D*_2_) necessary to reduce an *x*% cell viability (*D*_1/2_) divided by the dose necessary as a single-agent to generate reduction of a similar *x*% cell viability (*D*_*X*_)_1/2_. *D*_*1*_ olaparib, *D*_*2*_ TMZ. The line connecting the *X*- and *Y*-axis represents an additive effect (CI 1). Points left of the line are considered synergistic (CI < 1.0)

Olaparib (0.625; 1.25; 1.875 µM) and TMZ (10; 25; 50; 100; 250 µM) were combined in increasing dosages and drug synergy was examined by calculation of the CI. Similar to ES, combination treatment significantly decreased cell viability of DSRCT cells compared to the respective single-agent treatments, except for the 1.875-µM olaparib combinations and 10-µM TMZ combinations (Fig. 2a) (Engert et al. 2015; Gill et al. 2015; Stewart et al. 2014; Vormoor et al. 2017). Drug synergy assessment showed a synergistic interaction (CI < 1.0) between both compounds for all combinations, with a favorable DRI (Fig. 2b, Table 2). In line with cell viability effects, the level of drug synergy reduced in a dose-dependent manner with slight to moderate synergy seen for the combination of either 1.25 or 1.875 µM olaparib with 250-µM TMZ (Fig. 2b, Table 2). The dose-dependent decrease in drug synergy can be explained by the enhanced reduction in cell viability following high-dose single-agent treatment (Fig. 2a). Higher concentrations of the single agents lead to a significant reduction in cell viability compared to the control, allowing only a small window for the combination treatment to enhance these antitumor effects. The DRI was > 1 for each combination, indicating that the dosage of both drugs can be reduced in a combination treatment to elicit a similar effect as the respective single-agent treatments. The DRI was higher for TMZ compared to olaparib for all combinations involving ≤ 100-µM TMZ (except in the 0.625-µM olaparib combination), suggesting a potentiating effect of olaparib on TMZ. Similar to the CI, DRI showed a dose-dependent reduction, explained by the high single-agent effects. The highest efficacy (fraction of cell viability affected (FA value): 0.820) with the highest level of drug synergy (CI 0.457) was observed for the 1.25-µM olaparib and 100-µM TMZ combination treatment (Table 2).

**Table 2:** FA-, CI- and DRI values for olaparib and TMZ combination treatment

Olaparib (µM)	TMZ (µM)	FA value (mean ± SD)	CI	DRI (O; T)
0.625	10	0.477 ± 0.09	0.590	(1.83; 22.6)
	25	0.550 ± 0.09	0.520	(2.35; 10.7)
	50	0.679 ± 0.02	0.405	(3.72; 7.31)
	100	0.799 ± 0.04	0.348	(6.33; 5.26)
	250	0.833 ± 0.04	0.547	(7.67; 2.40)
1.25	10	0.679 ± 0.06	0.564	(1.86; 36.6)
	25	0.766 ± 0.04	0.425	(2.69; 18.8)
	50	0.792 ± 0.03	0.425	(3.05; 10.3)
	100	0.820 ± 0.04	0.457	(3.55; 5.69)
	250	0.833 ± 0.04	0.678	(3.83; 2.40)
1.875	10	0.765 ± 0.03	0.582	(1.78; 46.9)
	25	0.802 ± 0.04	0.513	(2.15; 21.3)
	50	0.799 ± 0.05	0.569	(2.11; 10.5)
	100	0.813 ± 0.05	0.620	(2.28; 5.54)
	250	0.819 ± 0.04	0.862	(2.37; 2.28)

*FA-value* fraction of cell viability affected by treatment, *SD* standard deviation, *CI* combination index, *DRI* dose reduction index, *O* olaparib, *T* temozolomide, *CI between 0.7–0.9* moderate to slight synergism, *CI between 0.3–0.7* synergism

### Olaparib and low-dose TMZ combination treatment

**Fig. 3:**
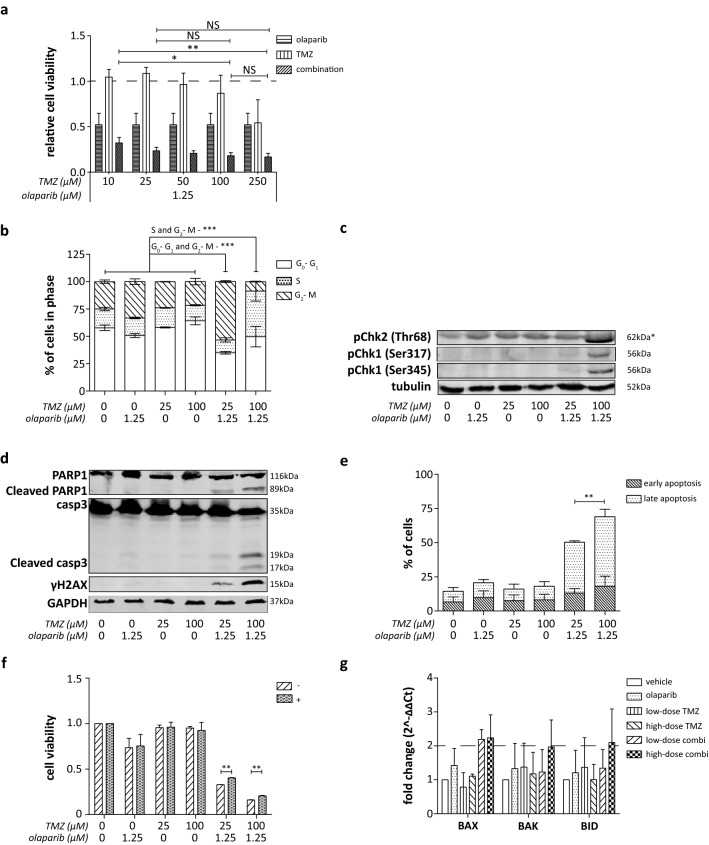
Low-dose olaparib and TMZ combination treatment in DSRCT cells. **a** Relative cell viability following 120 h simultaneous combination treatment of olaparib (1.25 µM) with increasing concentrations TMZ (10; 25; 50; 100; 250 µM). Dotted line represents the cell viability of vehicle-treated cells. **b** Cell-cycle arrest, **c** pChk1/2 expression (*: a-specific antibody binding at ~ 65 kDa), **d** PARP1 and caspase-3 (casp3) cleavage, DNA damage (γH2AX) and **e** induction of early and late apoptotic cells following 24-h treatment of low-dose (25 µM) and high-dose (100 µM) TMZ combined with olaparib (1.25 µM) in JN-DSRCT-1 cells. **f** Relative cell viability following 120-h single-agent olaparib (1.25 µM), TMZ (25 or 100 µM) and simultaneous low-dose and high-dose combination treatment in absence (−) and presence (+) of the pan-caspase inhibitor zVAD.FMK. **g** Pro-apoptotic protein BAX, BAK and BID mRNA expression following 24-h vehicle, single-agent olaparib (1.25 µM), TMZ (25 or 100 µM) and simultaneous low-dose and high-dose combination treatment. Dotted line represents the twofold change threshold. *p value < 0.05, **p value < 0.01, ***p value < 0.001

The potentiating capability of olaparib on TMZ efficacy was further examined by combining low-dose TMZ (10; 25 µM) with 1.25-µM olaparib (Fig. 3a). No difference in the effect on cell viability could be observed for the combination treatment of 1.25-µM olaparib and 25-µM TMZ compared to the combinations using 100-µM or 250-µM TMZ. The effect of combination treatment was not significantly different between 100-µM and 250-µM TMZ combination treatment, and all subsequent experiments were, therefore, performed using 1.25-µM olaparib with 25- or 100-µM TMZ as representative “low-dose” and “high-dose” combination treatment, respectively.

Similar to the findings of Engert et al*.,* low-dose combination treatment induced a G_2_-M phase arrest. Whereas, high-dose combination treatment induced a strong S-phase arrest (Fig. 3b). In line with the cell-cycle arrest, the activity of cell-cycle checkpoints 1 and 2 (pChk1/pChk2) increased following high-dose combination treatment (Fig. 3c/Fig. S2a–c). In addition, PARP cleavage, caspase-3 cleavage, DNA damage and apoptosis were induced following combination treatment. High-dose combination treatment led to significantly higher level of apoptosis, caspase-3 cleavage and DNA damage compared to low-dose combination treatment (Figs. 3d, e/S3d–f). Nevertheless, low-dose combination treatment still induced slight PARP and caspase-3 cleavage, DNA damage, and apoptosis (~ 50% of cells) following 24-h treatment (Figs. 3d, e/S3d–f).

Pan-caspase inhibition only slightly recovered the reduction in cell viability following both low-dose and high-dose combination treatment, suggesting a caspase-independent mechanism behind the observed apoptosis (Fig. 3f). Engert et al. showed a dependency on proteasomal degradation of the anti-apoptotic protein Mcl-1 with an increase in BAX/BAK activity. In line with these findings, gene expression of the pro-apoptotic proteins BAX, BAK and BID showed a  ≥ 2-time fold change following high-dose combination treatment (Fig. 3g).

Overall, these data show that olaparib is capable of potentiating TMZ efficacy and, in particular, combination treatment using low concentrations of both olaparib and TMZ is of interest for further in vivo and clinical evaluation.

### In vivo antitumor effects of olaparib and TMZ combination treatment in a DSRCT model

**Fig. 4:**
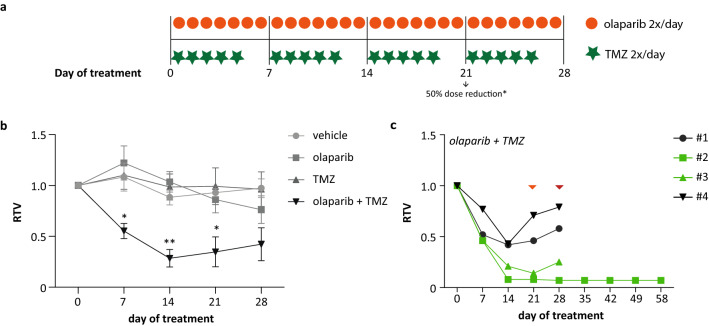
In vivo antitumor effects of low-dose olaparib and TMZ combination treatment in a DSRCT model. **a** Treatment schedule for the in vivo olaparib (50 mg/kg) and TMZ (25 mg/kg) combination treatment in mice bearing subcutaneous JN-DSRCT-1 tumors. *50% dose reduction for 2 out of 4 combination-treated mice was conducted on day 21 and treatment was continued for another 7 days. **b** Relative tumor volume (RTV) of the vehicle, single-agent (olaparib and TMZ) and combination treatment group at days 0, 7, 14, 21 and 28 of treatment. **c** RTV of the individual combination-treated mice at days 0, 7, 14, 21, 28, 35, 42, 49 and 56 of treatment. Green symbols represent the two mice given a 50% dose reduction following day 21 of treatment. Orange arrow indicates the moment of dose reduction. Red arrow indicates the moment of either the end of the experiment or treatment withdrawal. RTV is presented as mean ± SEM. *p value < 0.05, **p value < 0.01, ***p value < 0.001

In continuation of our in vitro data, we performed an in vivo assessment of olaparib and TMZ combination treatment in a JN-DSRCT-1-based mouse model. Olaparib and TMZ were administered twice daily for 7 and 5 days, respectively, during four cycles (Fig. 4a). The dose used (25 mg/kg) is equivalent to a human dose of 75 mg/m^2^. We administered the mice twice daily; whereas, patients receive TMZ once daily. However, the daily dose does still not exceed the 200 mg/m^2^ dose which is given to patients. In the clinic, they normally treat patients only for the first 5 days of a 28-day cycle. The mice have been treated in 4 cycles of 5-day treatments with only 2-day rest. We used this scheme since we have seen in another sarcoma model with comparable effectivity that treating mice for the first 5 days of a 28-day cycle showed tumor progression at day 10 (unpublished data). Tumor volume was significantly reduced in the combination group compared to the respective single-agent treatments and the vehicle group at days 7, 14 and 21 (Fig. 4b). This difference was no longer significant at day 28 due to a gradual decrease in tumor volume following olaparib single-agent treatment and a slight increase in tumor volume for the combination treatment group (Fig. 4b). The increase in tumor volume for the combination-treated group was most likely the result of tumor swelling due to intratumoral hemorrhage. Mice 1 and 4 of the combination group show a clear increase in vessel diameter and a higher level of intratumoral hemorrhage compared to the vehicle, olaparib and TMZ treatment groups (Fig. S3). One animal of the combination group was unfortunately prematurely taken from the experiment due to technical difficulties, resulting in *n* = 4 for this group. Single-agent treatments did not significantly affect the tumor volume compared to the vehicle group and can therefore be considered to be administered in low-dose concentrations (Fig. 4b). Grignani et al*.* recently described clinical efficacy of the combination of olaparib and trabectedin in a bone and STS study population using dosages of the individual drugs below the approved doses (Grignani et al. 2018). This shows that low-dose concentrations used in combination treatment can indeed potentiate the single-drug effects and induce clinically relevant effects. In addition, PARP-based combination treatment is not limited to TMZ or trabectedin. Stewart et al*.* showed complete and durable responses to the triple-combination treatment of a PARP inhibitor, topoisomerase I inhibitor irinotecan (IRN) and TMZ in in vivo ES models (Stewart et al. 2014).

### Treatment-related adverse effects in vivo

In the current study, we used olaparib, a PARP inhibitor with intermediate trapping capacities, for in vivo combination treatments (Murai et al. 2014). In addition to the FDA approval of olaparib for the treatment of breast cancer and ovarian cancer, we selected olaparib because Hopkins et al*.* showed that the tolerability of a PARP inhibitor and TMZ combination might be inversely correlated with the trapping capacities of the PARP inhibitor (Hopkins et al. 2015)*.* The choice of olaparib was made in an attempt to find a combination that has the potential to be tolerable and to enhance antitumor effects.

Vehicle and single-agent treatment groups did not experience treatment-related toxicity and combination treatment was well tolerated up to 3 cycles (day 21). At day 21, two mice with a near complete response to combination treatment (RTV = 0.08 and 0.21) showed treatment-related toxicity (Fig. 4c). In an attempt to reduce treatment-related toxicity, the dosages were reduced by 50% and the mice were treated for another 7 days until the end of the experiment. Dose reduction led to the recuperation (measured in activity) of 1/2 animals within 4 days. The second affected animal did not show a complete recuperation, although dose reduction did lead to health stabilization (data not shown). Necropsy did not show any toxicity-related alterations to the vital organs. A potential explanation for the level of toxicity seen in these two mice could be their general health, measured in body weight, at the start of the experiment. The affected mice showed a lower bodyweight at the time of injection (~ 23 g) and less growth in the first 14 days of treatment compared to their 2 counterparts in the same treatment group (~ 25 g) (Fig. S4). The mice with a higher body weight showed no signs of toxicity throughout treatment, did not need dose reduction, had an average tumor volume reduction of ~ 32% (21–42%), and showed a high level of intratumoral hemorrhage and tissue damage (Figs. 4c/S3). Moreover, one mouse with a near complete tumor reduction (RTV = 0.08) was taken off treatment at day 28 to assess response duration. Tumor growth was inhibited up to 28 days post-treatment withdrawal and within 7 days, treatment-related adverse effects (measured in body weight and activity) were no longer present (Figs. 4c/S4). Stewart et al*.* described a similar level of toxicity in an ES model. CD1-nude mice bearing orthotopic ES tumors showed toxicity following a phase I olaparib, TMZ and IRN combination treatment, which could be reduced by decreasing dosages by 50% without affecting treatment efficacy. Olaparib combination treatment, compared to talazoparib combination treatment, was better tolerated and the necessary dose reduction was limited to 50% compared to 70% with talazoparib (Stewart et al. 2014). A potential alternative to olaparib is given by O’Connor et al*.* Examination of the second-generation olaparib-derived PARP inhibitor AZD2461 combined with TMZ in a breast cancer model showed better tolerability in vivo compared to olaparib combination treatment (Oplustil O'Connor et al. 2016). Recently, the in vitro antitumor effects of AZD2461 combined with ionizing radiation (IR) were described for rhabdomyosarcoma cell lines, showing a significant increase in antitumor effects compared to IR alone (Camero et al. 2018). The effects of AZD2461 have, however, not yet been evaluated in in vivo sarcoma models.

The level of response in vivo does suggest that olaparib and TMZ combination treatment could be a potential treatment option for DSRCT patients. Current clinical trials of PARP inhibitor and chemotherapy combination treatment have resulted in hematological toxicities (Alecu et al. 2018; Zhou et al. 2017). The recent paper of Grignani et al*.,* however, showed clinical efficacy of combined treatment with olaparib and alkylating agent trabectedin combination treatment in a subset of bone and STS patients with manageable toxicities using dosages below the approved single-agent dose. This suggests that clinical trials using low dosages of olaparib and chemotherapy should be further evaluated since they might reduce toxicity to a manageable state and could lead to clinical responses in otherwise incurable patients, such as DSRCT patients.

### Preclinical DSRCT models

Here, we describe olaparib and TMZ combination treatment efficacy in a single preclinical DSRCT model, based on the lack of availability of other cell lines. In contrast to ES, preclinical models for DSRCT are scarce and JN-DSRCT-1 is currently the only established cell line (Nishio et al. 2002). The rarity of DSRCT hinders the development of more preclinical models. Moreover, the available primary tumor material is often excessively used for diagnostic purposes, preventing further access to the tissue for preclinical research. In addition, tumor tissue available for the development of preclinical models is often collected following intensive chemotherapy regimens, possibly altering the initial characteristics of the tumor tissue. Nevertheless, the lethality of DSRCTs, despite intensive chemotherapy, shows the unmet need for novel treatment options for this sarcoma subtype. International collaborations will be necessary to increase the availability of DSRCT tumor tissue, to foster the development of preclinical DSRCT models and to boost further preclinical research towards a better understanding of the pathogenesis of this complex disease and discovery of novel treatment options.

## Conclusion

DNA repair is essential to maintain genomic stability and cellular survival. Inhibition of DNA repair proteins was shown to enhance antitumor effects of chemotherapeutic agents in various models in a preclinical setting, and has recently shown encouraging clinical results (Baz et al. 2016; Grignani et al. 2018; Han et al. 2018; Kashyap et al. 2018). DSRCTs are currently almost always incurable. Despite initial favorable responses to ES-based multimodal treatment and second-line treatment, nearly all patients will eventually relapse. The rarity of DSRCTs makes research into this sarcoma subtype difficult, and there is an unmet need for novel treatment options. In the current study, we show for the first time that DSRCT tumor tissue shows a high level of PARP1 and SLFN11 expression, that DSRCT cells have a similar sensitivity profile to the PARP inhibitor olaparib as previously observed in ES cells and that combination treatment of olaparib with the alkylating agent TMZ leads to drug synergy and enhanced antitumor effects in vitro and in vivo. Moreover, the antitumor effects were already observed using low drug dosages. We consider our data of importance for the future treatment of DSRCT patients and suggest the inclusion of this patient group in current and future clinical trials addressing PARP-based combination treatments, including, but not limited to, the alkylating agent TMZ. In addition, the observed potentiating effect of olaparib on TMZ efficacy suggests that in particular, low-dose drug combinations are of interest for further clinical evaluation.

## Electronic supplementary material

Below is the link to the electronic supplementary material.

Supplementary file1 (PDF 612 kb)

## Abbreviations

DSRCT: Desmoplastic small round cell tumor
ES: Ewing sarcoma
PARP: Poly(ADP-ribose) polymerase
SLFN11: Schlafen-11
SSB: Single-stranded breaks
STS: Soft tissue sarcoma
TMZ: Temozolomide
