# Supplementary material for: Olaparib and temozolomide in desmoplastic small round cell tumors: a promising combination in vitro and in vivo
Source: J Cancer Res Clin Oncol. 2020 Apr 11;146(7):1659–70. doi: 10.1007/s00432-020-03211-z (PMC7256072; doi:10.1007/s00432-020-03211-z)
Supplement: Supplementary file 1 — Supplementary file1 (PDF 612 kb) [file 432_2020_3211_MOESM1_ESM.pdf]

Supplementary files

Olaparib and Temozolomide in Desmoplastic Small Round Cell Tumors, a promising combination *in vitro* and *in vivo*.

Anke E.M. van Erp[a], Laurens van Houdt[a], Melissa H.S. Hillebrandt-Roeffen[a], Niek F.H.N. van Bree[a], Uta E. Flucke[b], Thomas Mentzel[c], Janet Shipley[d], Ingrid M.E. Desar[a], Emmy D.G. Fleuren[e], Yvonne M.H. Versleijen-Jonkers[a], Winette T.A. van der Graaf[a,f]

[a]Department of Medical Oncology, Radboud University Medical Center, P.O. Box 9101, 6500HB Nijmegen, The Netherlands

[b]Department of Pathology, Radboud University Medical Center, P.O. Box 9101, 6500HB, Nijmegen, The Netherlands

[c]Dermatopathology Bodensee, Friedrichshafen, Germany

[d]Sarcoma Molecular Pathology Team, Divisions of Molecular Pathology and Cancer Therapeutics, Institute of Cancer Research, London, United Kingdom

[e]Children's Cancer Institute Australia, Lowy Cancer Research Centre, University of New South Wales, Sydney, NSW, Australia

[f]Department of Medical Oncology, The Netherlands Cancer Institute-Van Leeuwenhoek, 1066 CX Amsterdam, The Netherlands

Corresponding author:

Yvonne M.H. Versleijen-Jonkers

Department of Medical Oncology (internal postal code: 452)

Radboud University Medical Center

P.O. Box 9101

6500 HB Nijmegen, the Netherlands

Phone: 0031-(0)24-3618897

Fax: 0031-(0)24-3615025

E-mail: yvonne.versleijen-jonkers@radboudumc.nl

ORCID: 0000-0001-7625-0505

Table S1 **Forward (FW) and reverse (RV) primers pro-apoptotic proteins BAX, BAK and BID for RT-qPCR**

Primer	Sequence
Bax FW	TCAGGATGCGTCCACCAAGAAG
Bax RV	TGTGTCCACGGCGGCAATCATC
Bak FW	TTACCGCCATCAGCAGGAACAG
Bak RV	GGAACTCTGAGTCATAGCGTCG
Bid FW	TGGGACACTGTGAACCAGGAGT
Bid RV	GAGGAAGCCAAACACCAGTAGG

Fig. S1

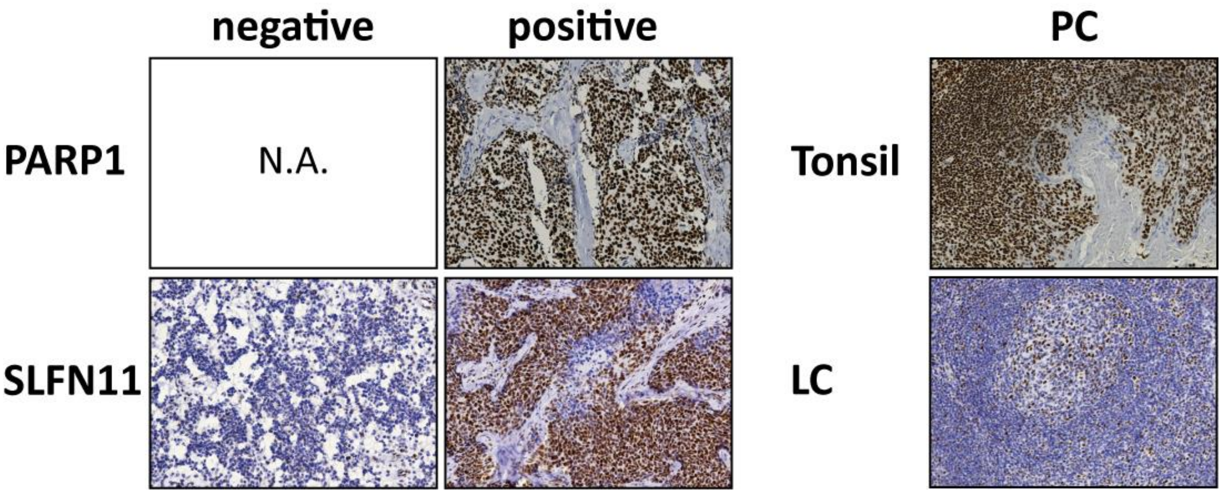

Figure S1 PARP1 and SLFN11 expression in DSRCT tumor tissue.

An example of negative and positive PARP1 and SLFN11 expression in DSRCT tumor tissue assessed by immunohistochemistry (IHC). All DSRCTs tumors were positive for PARP1 expression. Tonsil and lymphocytes (LC) served as positive control (PC) for PARP1 and SLFN11, respectively. N.A.: not applicable. Images were taken at 20x magnification.

S2

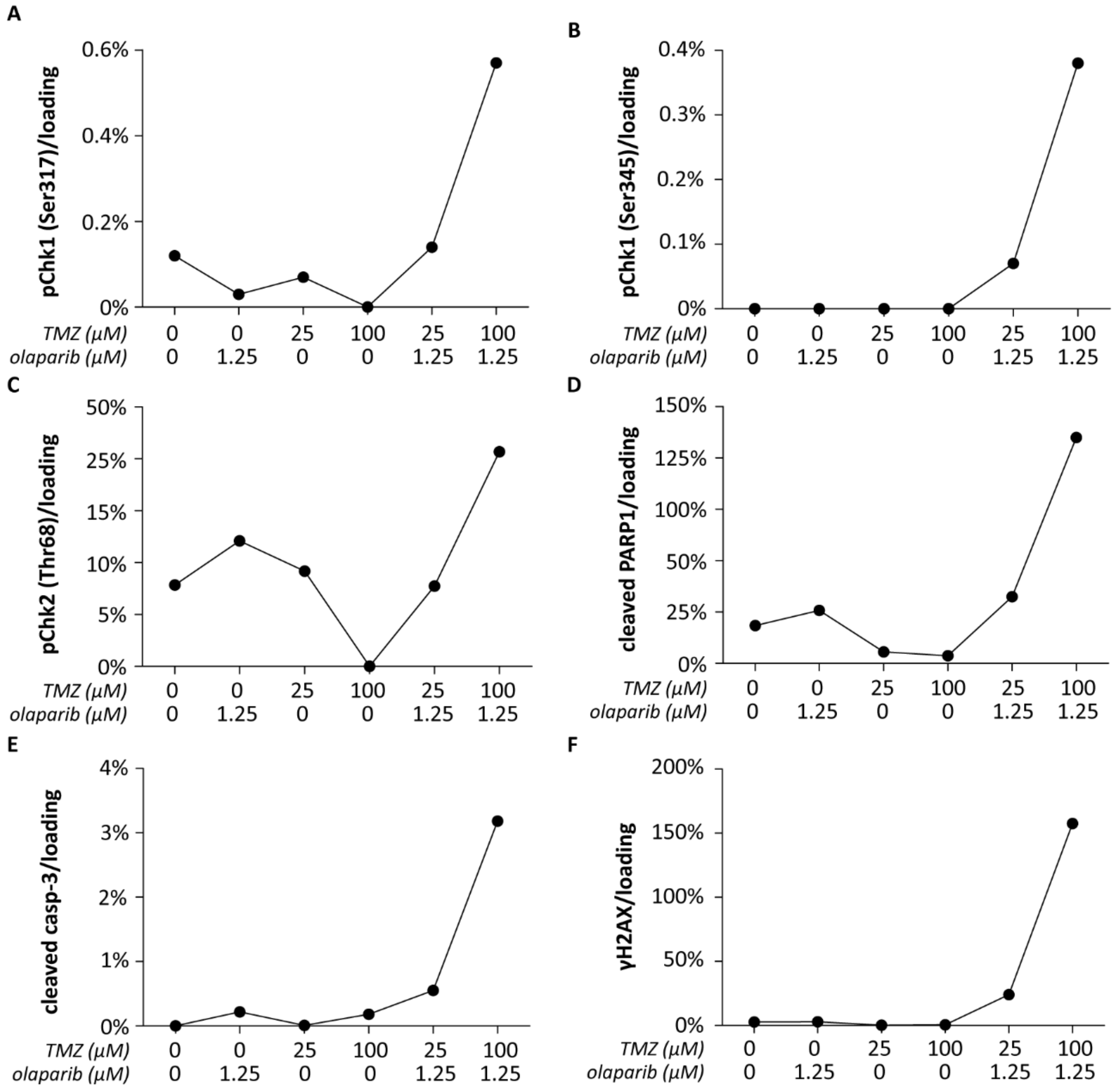

Figure S2 Quantification of pChk1/2, PARP1 cleavage, caspase 3 cleavage and γH2AX expression in JN-DSRCT-1 (a) Quantification of pChk1 (Ser317), (b) pChk1 (Ser345), (c) pChk2 (Thr68), (d) PARP1 cleavage, (e) caspase-3 cleavage and (f) γH2AX expression following 24h single-agent olaparib (1.25µM), TMZ (25 or 100µM) and low-dose and high-dose combination treatment in JN-DSRCT-1. Cleaved PARP1 and pChk1/2 expression is given as a percentage of the loading control.

S3

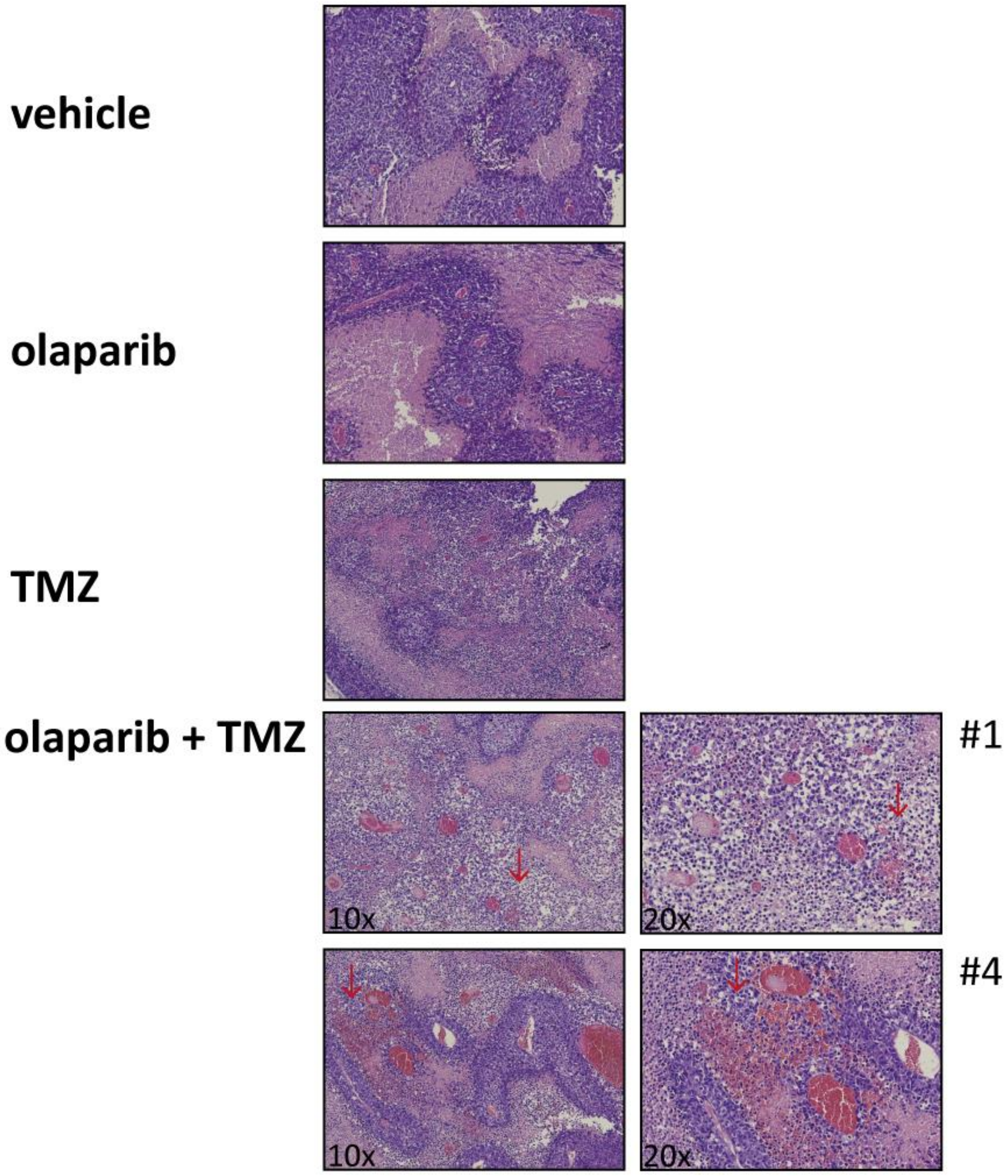

Figure S3 Blood vessels and intratumoral hemorrhage in *in vivo* tumors post-treatment
A representative image of a tumor containing blood vessels of the vehicle, olaparib and TMZ treated groups at 10x magnification. Representative images of the tumor of mice #1 and #4 of the olaparib and TMZ combination treated group showing dilated vessels and intratumoral hemorrhage (red arrow) at both 10x and 20x magnification. Mice were treated for 28 days and images show H&E staining.

S4

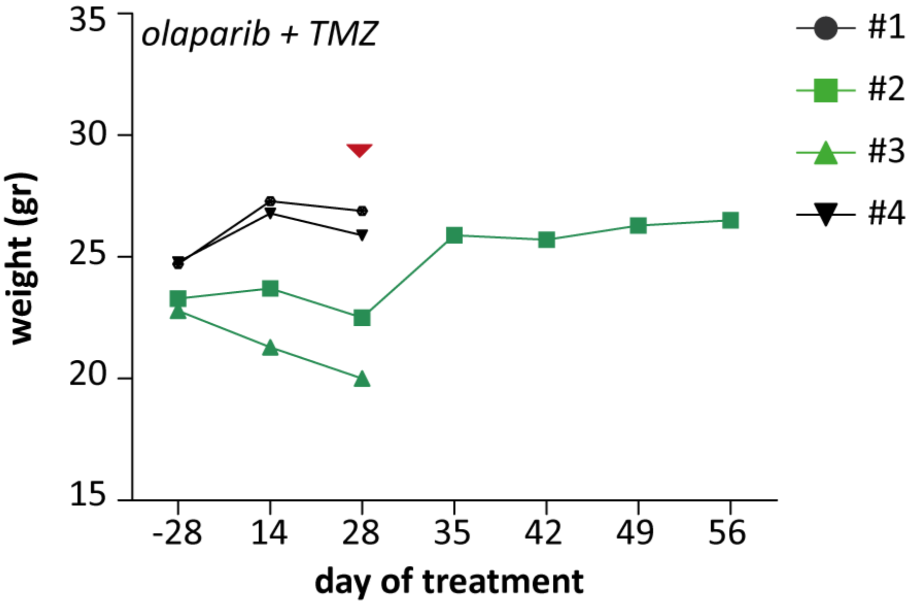

Figure S4 Weight of mice in combination treatment group
Weight of the mice at the day of subcutaneous cell injection (-28), day 14 and day 28 of treatment. Treatment-related toxicity observed in 2 out of 4 mice (green lines). One mouse (green squares) was kept in the experiment for another 28 days without treatment to assess health stabilization and durability of treatment response. Red arrow indicates either the end of the experiment or treatment withdrawal.
